# Psychomotor skills development for Veress needle placement using a virtual reality and haptics-based simulator

**DOI:** 10.1007/s11548-021-02341-0

**Published:** 2021-03-12

**Authors:** Chiara Di Vece, Cristian Luciano, Elena De Momi

**Affiliations:** 1 Department of Electronics, Information and Bioengineering (DEIB), Politecnico di Milano, Milan, 20133 Italy; 2 Department of Bioengineering and Simulation and Integrative Learning Institute, University of Illinois at Chicago, Chicago, 60607 (IL) USA

**Keywords:** Surgical simulation, Haptics, Virtual reality, Training, Psychomotor skills, Veress needle

## Abstract

**Purpose:**

Veress needle (VN) insertion, if not correctly performed, could cause severe injuries to intra-abdominal organs and vessels. Therefore, cognitive and psychomotor skills training is needed. Virtual reality (VR) and haptic technologies have the potential to offer realistic simulations.

**Methods:**

We developed a novel VR and haptic surgical simulator for VN insertion to teach trainees how to correctly puncture the abdominal wall, experiencing realistic tactile sensations throughout the simulation. The simulator allows for both procedural and realistic training. We released two different versions: the first using the OpenHaptics$^{\text {TM}}$ (OH) Toolkit and the second exploiting CHAI3D. We evaluated the learning effect using different performance indexes (time to perform the procedure, error in insertion angle, number of undesired contacts with organs) in an insertion task for both experienced urologists and students.

**Results:**

A general improvement of the chosen performance indexes was registered in the second repetition of the task for both groups. From the questionnaires, the simulator leveraging OH provides the trainee with a more precise haptic feedback, whereas the one exploiting CHAI3D allows them to perform the procedure more easily thanks to the better visualization of the virtual environment. The results proved that the participants appreciated both implementations, and the System Usability Scale (SUS) test resulted in a “good” usability.

**Conclusion:**

The haptics-based and VR simulator has shown the potential to be an important resource for the basic urological training in obtaining the pneumoperitoneum and improving the acquisition of the necessary psychomotor skills, allowing for extended and more effective training without compromising patient safety.

## Introduction

Improper access to the abdomen through Veress needle (VN) insertion is the cause of unique and life-threatening complications like injuries to intra-abdominal organs and vessels [1, 2]. The blind insertion and the firm reliance on the tactile sensation when inserting the VN through the different layers of the abdominal wall make this procedure risky for non-trained surgeons. The initial entry for abdominal insufflation is most frequently carried out at the umbilicus [3]. When the surgeon introduces the needle, the crucial step is the sensation of two distinct “pops” signaling that the tip is passing through the abdominal wall [4].

Since the surgeon needs to perform complex technical and cognitive tasks with tiny margin for error, it is difficult to acquire the required psychomotor skills through cognitive training only. The traditional Halstedian model of “see one, do one, teach one” [5] has been considered obsolete by many medical education experts mainly due to concerns regarding the connection between human error and patient mortality [6, 7]. Studies have pointed out that practice is the actual key for psychomotor skills acquisition [8] providing the trainees with physical and mental involvement in the learning process [9]. Students’ skills assessment is hugely subjective [10, 11]; however, over the last decade, simulation has offered a supervised environment in which strict skill evaluations and feedback help students develop clinical skills [12]. Principally, *computer-based simulation*, also known as virtual reality environments (VREs), overcomes the main limitations of the physical simulation (*e.g.,* *manikin-based simulation*) such as real-time feedback of trainees’ performances [13, 14]. Thus, VRE represents a potential solution for providing appropriate training in a reduced time and a safe environment before actually operating on a real patient [15, 16], a particularly crucial aspect for high-risk procedures [17].

In medical procedures, the sensation of touch is acknowledged as one of the essential physical senses [18]. An ideal simulator should create an immersive training environment that reproduces a specific surgery as faithfully as possible to provide the same stimuli present in the operating room (OR). Hence, the combination of virtual reality (VR) and haptic technology could be fundamental for the trainees, ensuring the active user’s participation in the simulation [19].

Several biomedical companies have developed different training systems based on haptics and VR to help trainees understand the necessary gestures fully and put them into practice [14]. Some examples of laparoscopic simulators are LapSim$^{\textregistered }$ by Surgical Since, Inc. (Sweden) and LAP Mentor$^{\text {TM}}$ by Simbionix (USA); they provide haptic feedback just for basic laparoscopic skills modules and quantitative assessment of trainees’ capabilities and improvements. The da Vinci Surgical Simulator by Intuitive Surgical (USA) is used for basic robotic skills training though lacking haptic feedback. Nonetheless, the unavailability of haptic feedback or, mostly, improper use, is one of the main drawbacks of available commercial simulators [20, 21].

Surgeons rely upon visual and haptic feedback, especially for needle insertions. To the best of our knowledge, the only available simulator for VN placement is a haptic and VR simulator prototype developed by Quanser Consulting Inc. and the University Health Network (Toronto, ON) [22]. The models incorporate tissues’ mechanical properties, allowing the user to feel the abdominal wall’s different layers. The authors did not evaluate the correct orientation and position of the VN’s tip, fundamental for teaching and assessment purposes.

Since the OR is not an ideal environment to practice and learn VN insertion due to recognized potential for injury, we evaluated the benefits of including VR and haptics to teach how to correctly puncture the abdominal wall without damaging the internal organs. The simulator allows for more effective surgical training and performance assessment by providing anatomically correct 3D visualization and realistic tactile feedback in a safe environment. We implemented the simulator using two different platforms: OpenHaptics$^{\text {TM}}$ (OH) [23] and CHAI3D [24], the main platforms for developing multisensory applications fast enough to be perceived by human senses as an interactive simulation [25].We then conducted a pilot study to verify the platform’s usability and assess the effectiveness of the proposed simulator.

The paper is organized as follows: “Methods” section presents the details about the two released simulators’ realization. “Results” section presents the main results obtained from the pilot study, discussed in “Discussion” section. Finally, “Conclusion” section presents our conclusions.

## Methods

Qualitative requirements for the simulator were defined with expert urologists’ support and the help of the Surgical Workflow Analysis [26]. They helped us identify the hidden challenges behind such a procedure. It should ensure the following specifications to meet the disadvantages of current training models and address the physicians’ needs: (i) anatomical realism, (ii) realistic haptic feedback, (iii) intuitive use for trainees, (iv) accurate measurements of the training outcomes, and (v) commercial viability and relevancy since this tool should be economically accessible for medical schools and convey the visual and tactile stimuli of a real operation.

### Graphics rendering

The 3D models of the abdominal organs were created from a patient’s anonymized dataset of CT images from DICOM Library [27] (voxel size: 0.59*x*0.59*x*0.5 *mm*, slice thickness: 1.46 *mm*, image size: 512*x*126*x*361). Using itk-SNAP$^{\text {TM}}$ [28], we pre-processed the images to eliminate artifacts and segmented the structures with a semi-automatic procedure based on *thresholding*. The obtained meshes have been carefully decimated to reduce the surface’s roughness and preserve its anatomical topology. Given the polygonal meshes, we cut one surface of skin, subcutaneous fat, rectus abdominis and linea alba to get just one front and one back face to not compromise the haptic feedback.

**Fig. 1:**
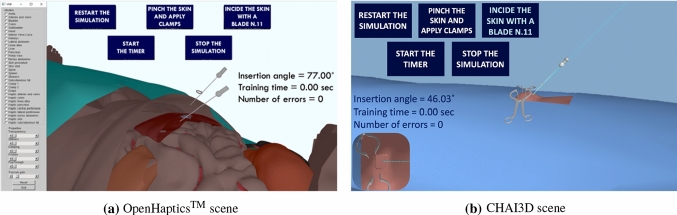
Obtained virtual scenes. **a** Cutting plane and frozen Veress needle in the OH application after insertion. On the left, the GUI to modify the models’ graphics and haptics parameters. **b** Insertion of the needle in the CHAI3D application. The line indicates the reference orientation of insertion

The obtained virtual scenes for both implementations are shown in Figure 1a and Figure 1b, respectively. By interacting with the buttons in the scene, the user can switch between three different virtual skin models to visually perceive the initial steps of the procedure: (i) start with the patient in the horizontal position, tilted of 15 degrees [29], (ii) pinch of the skin and application of two clamps to simulate umbilicus’ immobilization, and (iii) creation of an incision along the umbilicus’ crease.

The VN must be inserted through the umbilicus, pointing toward the anus, and forming a 45-degree angle with the patient’s body. Then, the trainee can freeze the VN in the position and orientation achieved by the haptic stylus and toggle the activation of a cutting plane. Thereupon, the shapes are clipped by a plane that changes its position and orientation according to the movements of the haptic stylus, visually reflected by the graphic cursor. Thus, the user can evaluate the VN proximity to the underlying organs. The graphical user interface (GUI) allows the user to modify the transparency values so that the user can further explore the body’s anatomical structures in the first phase of the training.

Besides, the implemented stereoscopic view provides the user with depth perception to better emulate the environment of the OR. The 3D monitor must support the side-by-side mode, and the trainee must run the application with proper 3D glasses.

In the CHAI3D application, we added a deformable portion to the skin, fat, linea alba, and peritoneum models to augment the scene’s fidelity, and a view from the top in the lower-left corner of the window.

### Haptics rendering

**Fig. 2:**
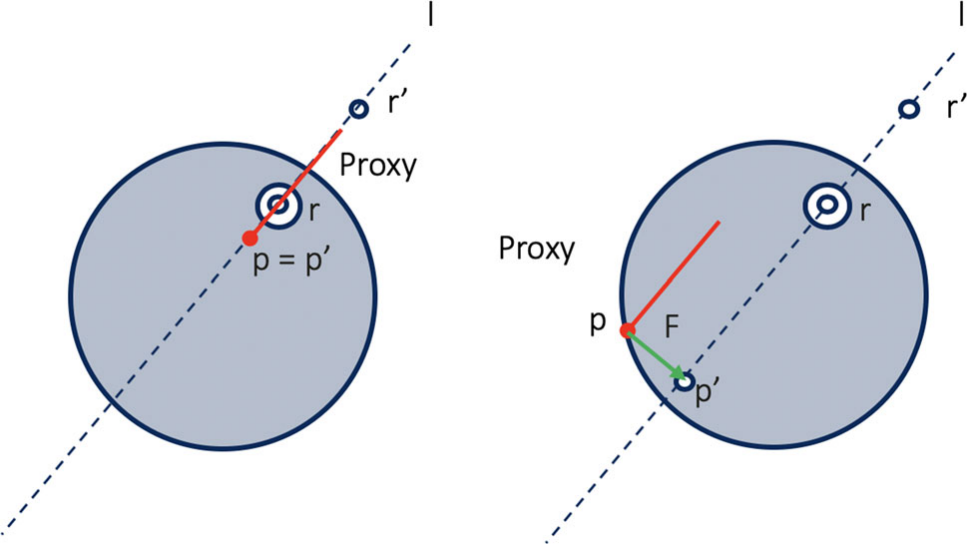
Fulcrum point effect

Even though certain technical limitations of the haptic device itself make it quite challenging to reproduce the exact tactile sensation obtained when performing the surgical procedure on real tissues, a cohort of ten volunteer, experienced urologists from University of Illinois at Chicago (UIC) was given the possibility of using the simulator for 30 minutes and tuning the parameters relative to tactile feedback based on their experience performing this surgical procedure on real patients, as done in [22]. They tune values (numbers between 0 and 1) for stiffness, damping, static and dynamic friction, and “pop-through”, *i.e.,* the force required to penetrate the abdominal structures with the VN, through the GUI, until the sensation was considered convincing. In CHAI3D, even though more limited in terms of haptic properties that can be assigned to meshes (damping and pop-through are not available), the values were tuned so that the perception remained as similar as possible to the OH simulator. We used a 3-degree-of-freedom (DoF) haptic device, the Touch$^{\text {TM}}$ 3D Stylus [30], to provide the force feedback from the organs. The sensation experienced when penetrating the tissues with a sharp instrument is emulated with the so-called *haptic fulcrum effect* (Figure 2). The surgeon can freely move the VN in the direction of insertion, back and forth, but not laterally. Given the insertion point *r*, the proxy position *p*, the proxy orientation $Q~=~(qw,~qx,~qy,~qz)$, the perceived force is $F~=~k~(p~-~p')$, where $p'$ is the projection of *p* onto *l*, *i.e.,* line passing for *r* and $r'~=~r~+~(qx,~qy,~qz)$.

When the skin is traversed, the user perceives a constant damping effect. As soon as the VN soaks into the underlying tissues’ surface, constant friction and increased damping are added until the user feels a give in-between linea alba and peritoneum and when reaching the peritoneal cavity to simulate the two “pops”. The collisions between the VN and the organs are detected using the proxy-based algorithm [31]. In CHAI3D, this is significantly sped up through the so-called *bounding volumes*. For the deformable objects, only impacts between one or more filling spheres and the proxy are considered [32].

### Performance evaluation

The simulator provides the trainees with quantitative feedback. The insertion results successful if performed with the proper orientation and without puncturing the underlying organs. Elaborated quantitative data are automatically saved and allow the user to track the improvements. In the surgical field, expert surgeons can be asked to choose metrics that they believe reflect surgical performance in the simulated surgical task, as proposed in [33]. Hence, urology instructors who routinely teach this surgical procedure defined the performance metrics:

- *Insertion error*: relative orientation of the skin and the VN; a correct needle orientation is mandatory to reduce the risk of injury for the nearest organs.
- *Duration of the task*: time interval, measured in seconds, from the start of the timer and its stop.
- *Number of mistakes*: number of undesired collisions between the virtual VN and virtual models of arteries and veins, colon, pancreas, and lateral peritoneum plus the number of insertions with wrong orientation.

### Study design

We recruited fourteen right-handed volunteers from both the European Institute of Oncology (IEO) in Milan (males, aged 38.43 ± 6.68) and Politecnico di Milano (62% females and 38% males, aged 29.63 ± 0.94). We performed a cross-center study to understand how subjects differ in achieving improvements, and perform an expertise-independent evaluation. To understand without biases both implementations’ pros and cons, we randomly divided each of the two groups (experienced urologists and students) into two separate subgroups. Two subgroups started the training with OH simulator, whereas the other two subgroups with the CHAI3D one. All subjects provided signed informed consent prior to the testing phase. Since none of the participants has any previous experience with this simulator, we offered a brief introductory orientation session. However, they were not allowed to practice before starting their session. Each participant was solicited to access the abdominal cavity twice, as done in [34], with the VN controlled by the haptic device stylus. The virtual patient is fixed in a prone position to make the tests reproducible and comparable. We asked them to:

1. Touch the buttons on the screen with the haptic cursor to visually go through the preliminary steps;
2. Start the timer;
3. Insert the virtual VN paying attention to the subtle “pop” sensation when piercing the linea alba and peritoneum, and carefully defining the needle angle at 45 degrees with respect to the patient’s body. A tolerance of ±5 degrees is acceptable;
4. Avoid undesired contacts;
5. Press button 2 on the haptic device to freeze the needle and perform self-assessment, as follows:
  - *Group 1* can manipulate a 3D clipping plane to crop out the virtual patient and reveal the internal anatomy and the inserted VN;
  - *Group 2* can make clamps, drape, and superior anatomical layers transparent to see the virtual needle inside the anatomy;
6. Stop the timer;
7. Repeat all the previous steps, now with better knowledge about the patient’s anatomy and more familiarity with the simulator.

Then, we asked each participant to complete:

- A specific questionnaire about their experience with the simulator consisting of 12 questions, either open or multiple-choice. The first questions aim at gathering information about participants’ familiarity with VN insertion and VR simulations. Questions from 3 to 11 are multiple-choice questions to rate participants’ experience with the simulator. We used the last questions to obtain further general feedback. The possible answers range from 1 (not at all) to 5 (extremely).
- A general System Usability Scale (SUS) questionnaire for subjectively assessing the new technology usability [35]. The respondent has to indicate the extent of agreement or disagreement with each statement on a 5-point scale.

We collected data to evaluate the surgical performance based on parameters presented in “Performance evaluation” section. Data were compared through a nonparametric statistical hypothesis Wilcoxon signed-rank test. A two-tailed p-value < 0.1 is considered significant. We present descriptive data in terms of median and quartiles.

## Results

### Haptics parameters setting

As explained in “Methods” section, the default values for the haptic properties are obtained by averaging the different values set for each organ by the ten urologists from UIC (Table 1).

**Table 1:** Default values of the haptic properties set at UIC

Model	Stiffness	Damping	Friction	Pop through
Bowel	0.02	0	0	0.01
Linea Alba	0.5	0.1	0.1	0.02
Pancreas	0.1	0	0	0.05
Peritoneum	0.2	0.05	0.35	0.05
Skin	0.4	0.1	0	0.05
Subcutaneous fat	0	0	0	0
Vasculature	0.2	0	0	0.2

### Quantitative evaluation

For the OH application, a statistically significant difference was found in terms of total time employed to perform the procedure (p < 0.001 for both groups), insertion error with respect to the reference one (p$_\text {surgeons}~<$ 0.1 and p$_\text {students}~<$ 0.05) and total number of errors (p < 0.001 for both groups). For the CHAI3D application, the total time shows p$_\text {surgeons}~<$ 0.1 and p$_\text {students}~<$ 0.001, whereas insertion error with respect to the reference shows p$_\text {surgeons}~<$ 0.05 and p$_\text {students}~<$ 0.001.

We computed the median values, and first and third quartiles for experienced urologists and students’ two attempts on both platforms (Table 2). All participants performed better on the simulator in their second attempt rather than in their first one on both platforms.

**Table 2:** Median values with first and third quartiles for performance indexes for attempts A and B on both platforms for both groups

Performance indicators	OpenHaptics$^{\text {TM}}$ **Application**				**CHAI3D Application**			
	Experienced Surgeons		Students		Experienced Surgeons		Students	
	*Attempt A*	*Attempt B*	*Attempt A*	*Attempt B*	*Attempt A*	*Attempt B*	*Attempt A*	*Attempt B*
Insertion angle	57	44	45.54	46.47	50.99	46.87	48.53	45.76
(degrees)	(42.67–59.54)	(43.86–45.37)	(37.08–52.26)	(42.81–47.74)	(43.31–57.84)	(45.67–48.555)	(40.85–50.15)	(44.96–46.56)
Relative error	12	1	7.26	2.74	7.58	1.87	5.16	1.09
(degrees)	(2.95–14.54)	(1–1.14)	(7.26–8.53)	(2.19–3.82)	(5.86–12.835)	(0.67–3.55)	(3.79–7.13)	(0.63–1.84)
Total time	83	61	69	51	64	60	64	52
(sec)	(79–84)	(60–70)	(69–74)	(50–60)	(60.5–72)	(53.50–61.50)	(60.5–68.5)	(51.50–57.00)
No. total errors	2	1	3	1	3	1	2	1
	(2–3)	(0–1)	(3–4)	(1–1)	(3–3.5)	(0.5–1)	(1.5–3.5)	(1–1)

**Fig. 3:**
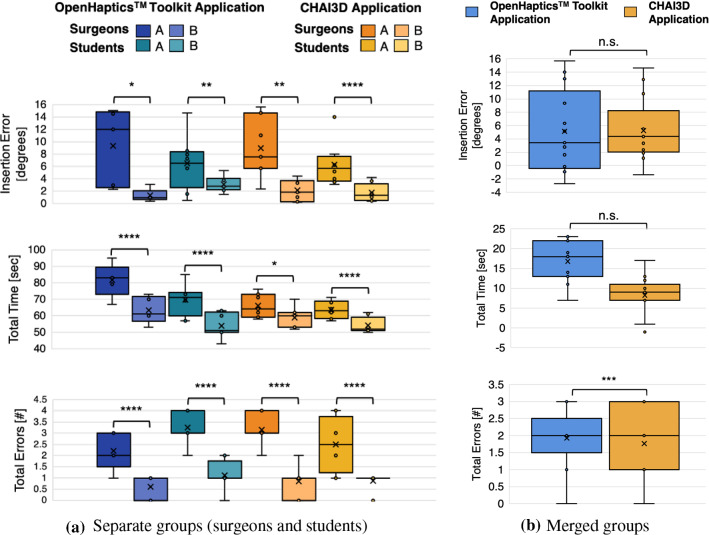
Boxplots of performance indicators for attempts A and B on OH (blue) and CHAI3D (orange) applications. The significance is reported on the boxplots using the stars: **** for $p < 0.001$, *** for $p<0.01$, ** for $p< 0.05$, * for $p<0.1$, n.s. otherwise. **a** Evaluation for separate groups. **b** expertise-independent evaluation (merged groups)

Then, we compared the two implementations in terms of gained improvements among the two attempts for both groups by computing the differences between the obtained values for each simulator. In both cases, the only remarkable difference is the reduction of the total employed time, more significant in the OH implementation (p$_\text {surgeons}~<$ 0.001 and p$_\text {students}~<$ 0.1, respectively). Finally, we performed an expertise-independent evaluation to understand which platform allows for the best improvements regardless of the subjects’ expertise. With merged groups as well, the only remarkable difference is the reduction of the total employed time, always more significant in the OH implementation (p < 0.005). The boxplots of performance indicator comparisons between OH and CHAI3D applications for separated and merged groups are reported in Figure 3a and Figure 3b, respectively.

### Qualitative evaluation

There was a general recognition of the application’s potential impact as a training tool for learning how to obtain pneumoperitoneum with VN insertion. However, the attitude to accept the technology as a training tool was different between the more experienced urologists and the students. Although recognizing its usefulness, the first ones were less willing to abandon the traditional approach; on the contrary, the students, despite being a novel technology, demonstrated an excellent willingness to accept the simulator as a training tool. It has been pointed out that the haptic feedback triggered when puncturing the skin and penetrating the fascia, as well as all the haptic properties related to the 3D models, were considered more convincing in the OH implementation, allowing for a more straightforward discrimination of the different abdominal layers. Conversely, even though the haptic feedback in the CHAI3D implementation resulted slightly less realistic, the visualization of the anatomy and the insertion of the virtual VN was more natural to perform thanks to the presence of the second camera with the top view.

We computed an initial global SUS score by averaging each participant’s scores. Hence, we obtained a SUS score for both groups and both platforms. We then merged the data from the two groups to understand which platform allows for the best improvement regardless of the subjects’ expertise. We obtained $SUS_\text {OH}~=~68.86~\pm ~1.29$ and $SUS_\text {CHAI3D}~=~70.29~\pm ~1.56$.

**Fig. 4:**
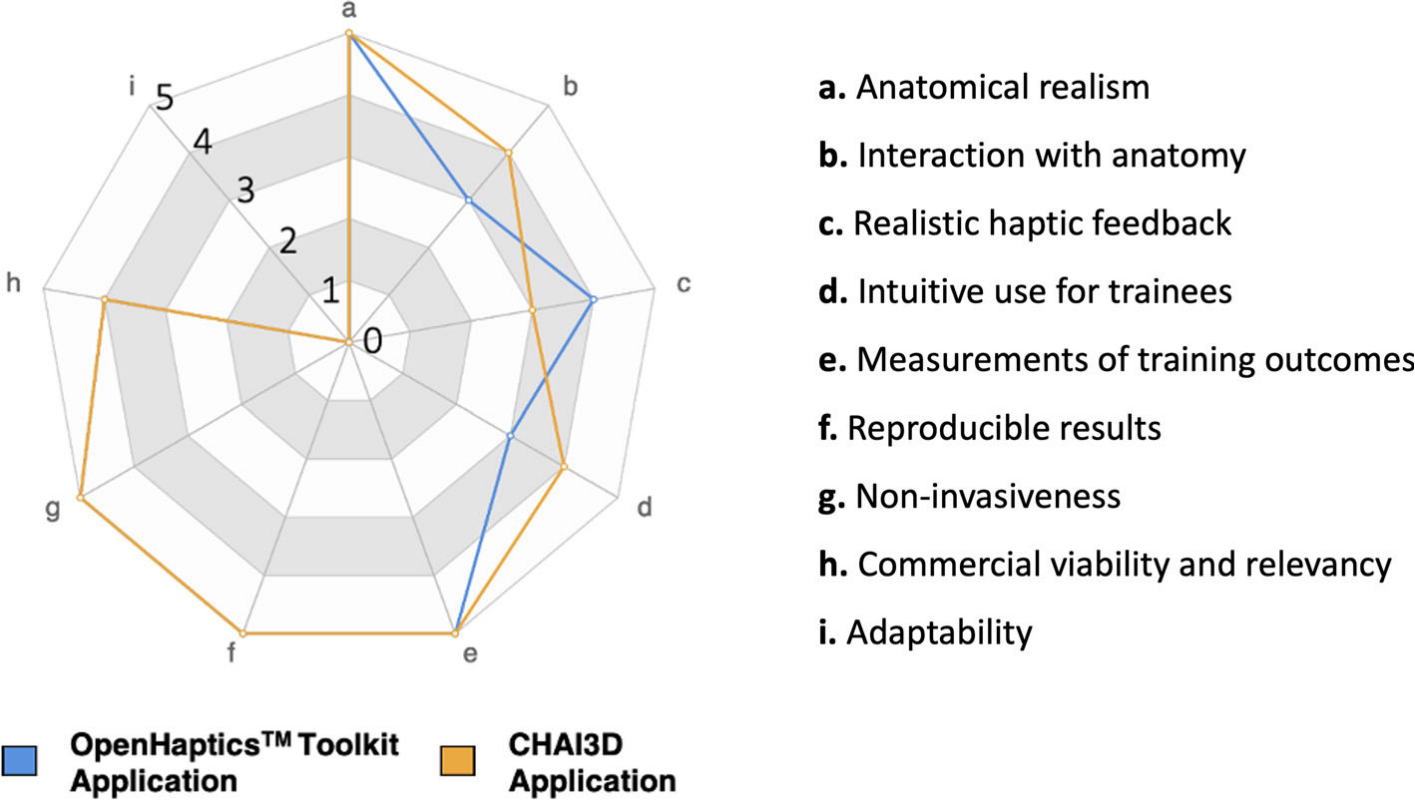
Solution features with respect to qualitative requirements

In Figure 4, we report the solution features with respect to the qualitative requirements of the simulator defined in “Methods” section.

## Discussion

In this pilot study, the participants’ overall goal was to decrease the error in the insertion angle from attempt A to attempt B without touching underlying organs in a shorter amount of time. Data relative to the performance indexes revealed a general improvement in the second repetition of the task on both the platforms and for both groups; all the medians of the performance indexes are lower in attempt B in both applications. The participants rapidly gained confidence with the haptic device and the simulator, and while in their first approach they seemed to be cautious, in the second one they performed the task faster, shortening the total time and attesting the intuitiveness of the training platforms. This is particularly evident in the case of students from Politecnico di Milano; the main reason is that even though they do not have in-depth knowledge about VN insertion or the anatomy, they do have more hands-on skills with haptic devices and simulators in general. The improvements in the insertion angle were lower in the CHAI3D application, in all scenarios; however, the relative angle between the skin and the virtual needle was nearer to the correct one even in the first attempt thanks to the better virtual scene visualization. On the contrary, the number of undesired interactions with organs not involved in the procedure was lower in the OH application; in this case, this may be due to the finer perception of the different layers of the abdomen. At the same time, a difference could also be noticed between the two groups: the experienced surgeons committed fewer errors due to these interactions with respect to the students, who have lower knowledge of anatomy. Considering the evaluation performed with the SUS, both systems have achieved an overall “good” usability [36] in all the considered scenarios.

Some feedback was given from expert urologists to improve the simulator’s content: i) improvement of visualization in the OH implementation and tactile sensation in CHAI3D one; ii) design of a dynamic application by considering the displacement of the organs due to respiration using a 4D CT scan; iii) inclusion of a VR headset to have a far-reaching visual of the virtual OR and of iv) an automated organ segmentation algorithm to allow for patient-specific simulations.

## Conclusion

In this paper, we presented a VR and haptic-based simulator for training in the blind and risky VN placement. Due to the difficulties in performing this step, the trainees are prevented from gaining experience and confidence in the surgery by only attending the OR.

Even though the early prototype of the haptic VN insertion simulator described in Sect. 1 appeared and felt pretty realistic, authors only evaluated stiffness and break force for the two different fascial layers and did not validate it. Besides, the simulator’s use can accelerate the training phase, reducing the learning time and overcoming the drawbacks of currently available solutions based on physical simulation. It integrates visual and haptic information on a patient’s 3D models of all abdomen’s organs extracted from a volumetric dataset. Before the implementation, we studied the surgical procedure in depth, thanks to available videos and consultations with experienced urologists who routinely perform and teach it. Two versions were implemented: one using OH, and the other CHAI3D; different from OH, this is an open-source platform, but with a lower development state [25]. The OH application shows better performance in terms of graphics framerate; thus, it can be easily adapted for less powerful machines. Questionnaires’ results proved the experiment participants appreciated it. Particularly, the first implementation allows for better haptic feedback when puncturing the different abdominal layers. The second one provides the users with a better graphical visualization, including tissue deformation. The simulator’s value was acknowledged for the training of urologists, who, in their residency, need to learn how to place the VN correctly inside the abdomen with a proper orientation without puncturing the underlying organs. Since this is a pilot study, further randomized clinical trials enrolling larger samples of trainees are necessary to increase the statistical significance of results and, therefore, validate the preliminary ones. We will investigate the transfer of skills achieved by using the simulator to real tasks, analyzing the improvements over a longer period, and considering additional performance metrics to draw concrete validation conclusions. Since there could be difficulties, both ethical and practical, in comparing surgical simulation directly to real surgery, particularly in novices’ training, we will consider conventional training techniques. Besides, it could be interesting to perform a biomechanical characterization of tissues with traction and tension tests on the models, as well as to use of a 6-DoF haptic device to provide force feedback based on both force and torque.
